# A Rare Case of Large Schwannoma of the Parapharyngeal Space

**DOI:** 10.1155/2018/9870937

**Published:** 2018-06-12

**Authors:** Nora Siupsinskiene, Irina Arechvo, Rimante Lapinskaite, Evaldas Padervinskis, Silvija Ryskiene, Saulius Vaitkus

**Affiliations:** ^1^Department of Otorhinolaryngology, Academy of Medicine, Lithuanian University of Health Sciences, Eiveniu 2, LT-50161 Kaunas, Lithuania; ^2^University of Klaipeda, Herkaus Manto 84, LT-92294 Klaipeda, Lithuania; ^3^Department of Ear, Nose and Throat Diseases, Republican Vilnius University Hospital, Siltnamiu 29, LT-04130 Vilnius, Lithuania; ^4^Raseiniu Hospital, Ligonines 4, LT-60127 Raseiniai, Lithuania; ^5^Department of Radiology, Academy of Medicine, Lithuanian University of Health Sciences, Eiveniu 2, LT-50161 Kaunas, Lithuania

## Abstract

Schwannoma originating from the peripheral nerves is a rare lesion of the parapharyngeal space. The special traits of the presented case included the following: the patient presented with slowly progressing dysphagia, speech difficulties, jaw numbness, and taste dysfunction. A dislocated lateral pharyngeal wall with mild inflammatory changes of the oropharyngeal mucosa was observed during pharyngoscopy. The radiological and histological characteristics of the neoplasm are consequently presented. Special emphasis is placed on the surgical treatment of the tumor.

## 1. Introduction

The parapharyngeal space is an inverted pyramid-shaped area of the deep tissues of the neck. The pyramid is based in the skull base, and its top extends to the greater horn of the hyoid bone. Clinically, the styloid process divides the parapharyngeal space into two segments. The prestyloid and poststyloid compartments are separated by the fascia of the tensor veli palatini muscle. The anterolateral prestyloid compartment contains the retromandibular portion of the deep lobe of the parotid, adipose tissue, small or ectopic salivary glands, a small branch of the trigeminal nerve supplying the tensor veli palatini muscle, the ascending pharyngeal artery, the pharyngeal venous plexus, and lymph nodes. The largest part of the posteromedial poststyloid compartment consists of fat. This space also contains the internal carotid artery and jugular vein, as well as cranial nerves IX–XII, the cervical sympathetic trunk, and lymph nodes.

Schwannoma is a relatively rare, slow-growing benign tumor that develops from a myelinated coating of the peripheral nerve [1]. Schwannoma may appear in any part of the body. The literature reveals that in 25 to 45 percent of cases, the tumor develops in the head and neck area, while it is rarely found in the parapharyngeal space [2]. Most tumors of the parapharyngeal space are benign and form from the salivary gland tissue [2, 3]. The tumors of neural origin are characterized by an insidious course and are consequently often delayed in diagnosis. The treatment depends on the size and location of the tumor [4].

The aim of this paper was to present an exceptionally rare and difficult case of large schwannoma of the parapharyngeal space possibly of the small branch of the mandibular nerve (V3) and to review the scientific literature.

## 2. Case Report

**Figure 1:**
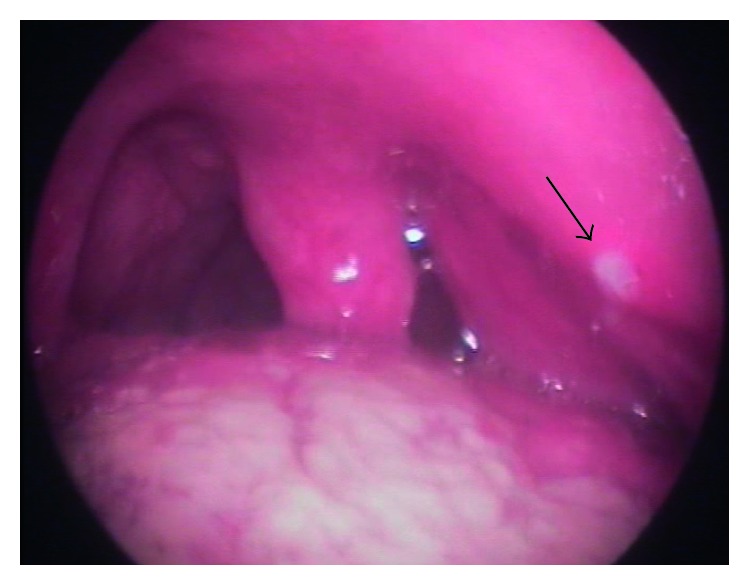
The oropharyngeal lumen is narrowed by a mass in the left parapharyngeal space. A small area of fibrin is visible at a previous puncture point (arrow).

A 32-year-old man was referred to the Lithuanian University of Health Sciences Kaunas Clinics Hospital with the symptoms of throat discomfort on the left side and dysphagia. The symptoms persisted for approximately 2 months. At arrival, the patient had no fever and there were no other signs of acute infection. Anamnestically, the patient was treated with antibiotics due to a suspected peritonsillar abscess on the left side for a period of 1 month. His left peritonsillar area was repeatedly punctured. However, only blood was obtained with a puncture. The prescribed antimicrobial therapy was not effective—dysphagia progressed, the patient started to report more speech difficulties, his lower jaw became numb, and taste dysfunction appeared. During pharyngoscopy, a dislocated lateral pharyngeal wall with mild inflammatory changes of the oropharyngeal mucosa was observed. The palate tonsil was displaced towards the uvula (Figure 1)

The fibronasolaryngoscopic investigation revealed that the left side of the nasopharynx was narrowed by a large mass covered with an intact smooth mucous membrane. No pathology was observed in the larynx—the color of mucosa was normal, and the vocal cords were mobile and smooth. No additional structures were seen. Neck lymph nodes could not be palpated.

**Figure 2:**
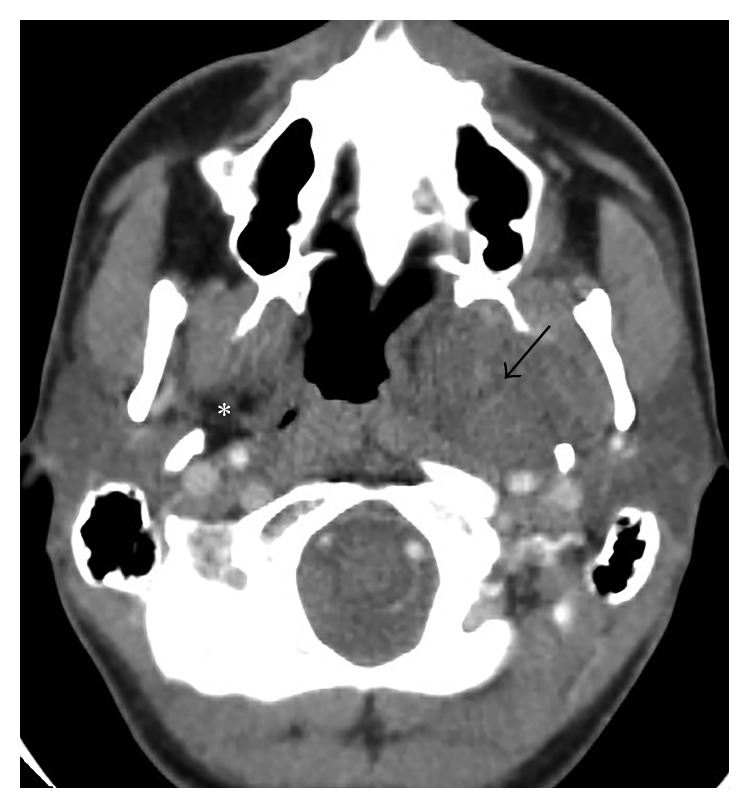
The contrast-enhanced axial CT scan shows the nonhomogeneous tumor within the left parapharyngeal space with a narrowing pharyngeal lumen (arrow). Note the normal fat contour in the right prestyloid compartment (asterisk).

Due to the suspected pharyngeal tumor, the patient underwent a contrast-enhanced computed tomography (CT) study, which showed a clearly limited, oval-shaped lesion in the left parapharyngeal space (Figure 2).

The size of the tumor was 4.2 × 3.3 × 6.7 cm. It was characterized by a nonhomogeneous structure with multifocal intratumoral hemorrhages of varying ages. The tumor encased the carotid arteries and the styloid process, while it stretched the pterygoid muscles on the left side and remodeled the pterygoid processes of the sphenoid bone. The medial part of the tumor pushed the palatal tonsil and uvula towards the centerline, as well as the root of the tongue to the front and the middle. Moreover, it significantly deformed the oropharyngeal and nasopharyngeal cavities. The upper part of the tumor ascended and tapered to the bone surface of the base of the skull and extracranial oval foramen. The lower pole of the tumor reached the submandibular salivary gland level and dislocated it slightly laterally.

**Figure 3:**
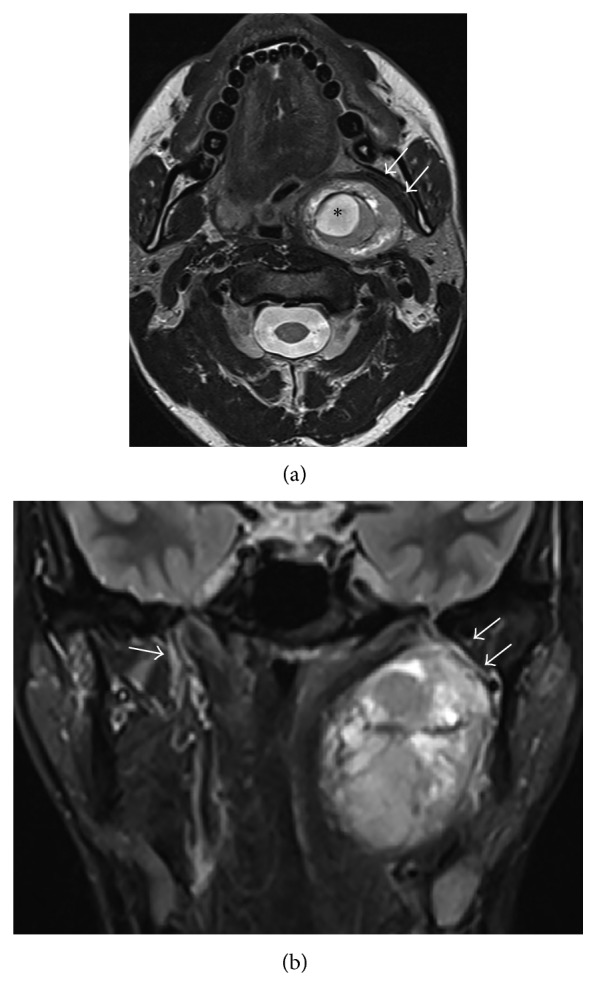
(a) Magnetic resonance imaging, axial projection. A large mass with cystic degeneration (asterisk) dislocating the root of the tongue is seen in the left parapharyngeal space. Stretched pterygoid muscles are shown with arrows. (b) Coronal projection shows the limits of the tumor (upper: the skull base; lower: the level of the submandibular salivary gland). Double arrow shows the laterally dislocated third branch of the trigeminal nerve on the left side. Note the normal proximal segment of the V3 on the right side (thin arrow).

To clarify the diagnosis, the patient underwent a magnetic resonance imaging (MRI) study. The study showed that due to its localization and tumor-specific features, the most likely diagnosis was schwannoma of the small branch of the mandibular nerve (V3) since a limited formation of specific localization with a component of cystic degeneration was found. Deformed from the medial part, the lateral pterygoid muscle with the V3 is shown in Figure 3.

**Figure 4:**
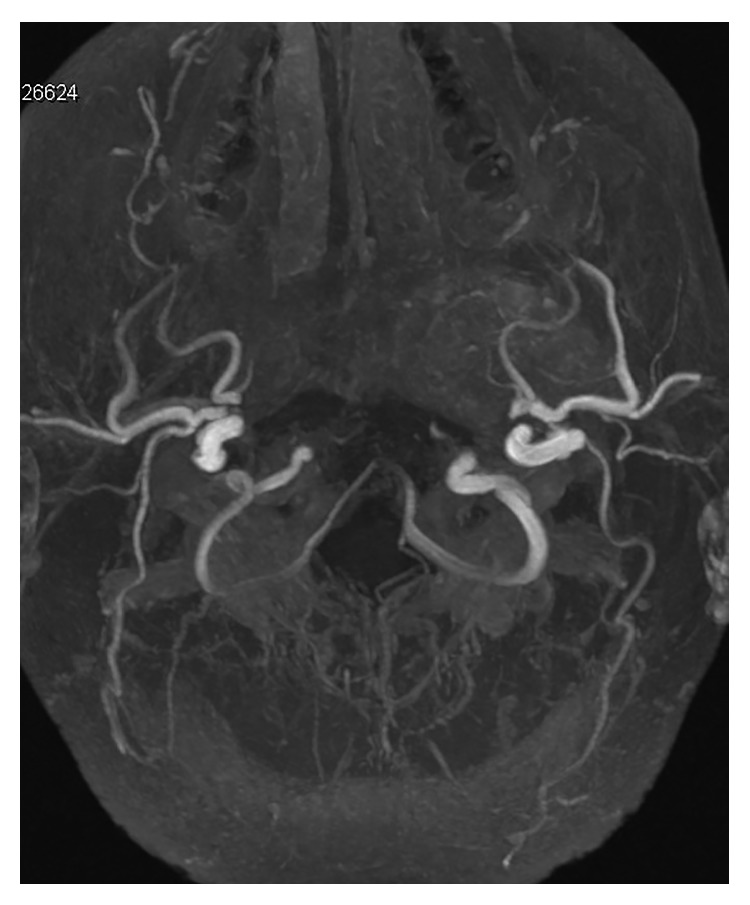
An angiographic study showed the tumor contour and contrasted tumor-feeding blood vessels—the ascending pharyngeal artery was the main vessel.

To determine a final diagnosis and plan a further treatment, a biopsy with histological verification of the tumor was performed. Under local anesthesia, a punch biopsy was carried out. A histological examination confirmed the diagnosis of schwannoma. Transoral removal of the tumor was planned. During an angiographic study prior to the surgery, the tumor-feeding blood vessels, of which the main vessel was the left ascending pharyngeal artery, were identified and embolized (Figure 4).

After the preparation of the patient, the schwannoma extirpation through a 5 cm incision at the left wall of the pharynx from the hard palate to the root of the tongue was performed under general anesthesia, while the widening of the oropharynx was done with a throat gag. The tissues were bluntly separated and the tumor capsule was reached, while the bloodstream of the surrounding area was externally disconnected. The tumor was removed in parts, thus reducing its volume. Later, it was totally removed with a capsule. No postoperative complications were observed. The wound healed by primary intention. The patient was discharged for further outpatient treatment on the sixth postoperative day. Antibiotic therapy with penicillin and painkillers was prescribed postoperatively.

**Figure 5:**
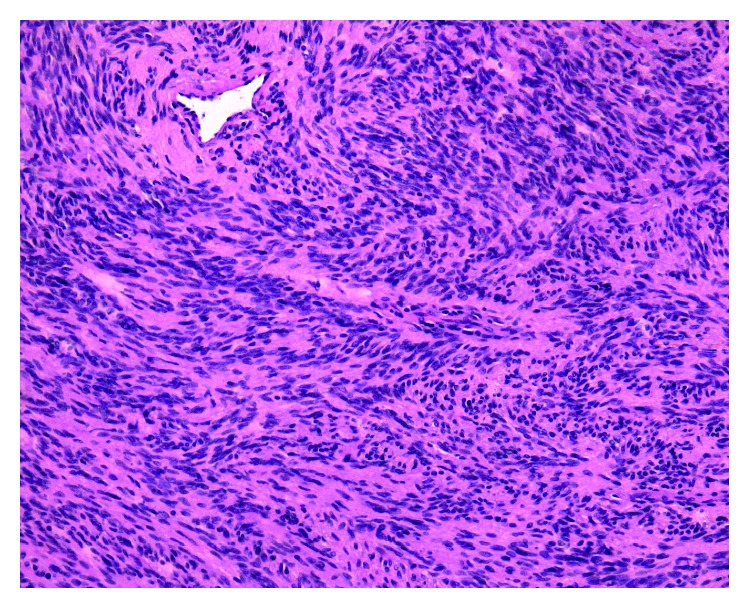
Histopathology showing monomorphic spindle cells with oval nuclei and eosinophilic cytoplasm positive for S100 calcium-binding protein P. The tumor cells formed palisade structures.

Histological examination of the operating material revealed a tumor that formed by monomorphic, mitotically inactive spindle-shaped cells with oval nuclei and an eosinophilic cytoplasm, which were positive for S100 calcium-binding protein P (Figure 5).

Cells were either fascicular or palisade in structure. Larger individual cells with large and irregularly shaped nuclei were found. Furthermore, thick-walled blood vessels, xanthoma accumulations of macrophages, and abundant groups of hemosiderophages with several foci of necrosis were also visible. The diagnosis of schwannoma was confirmed.

**Figure 6:**
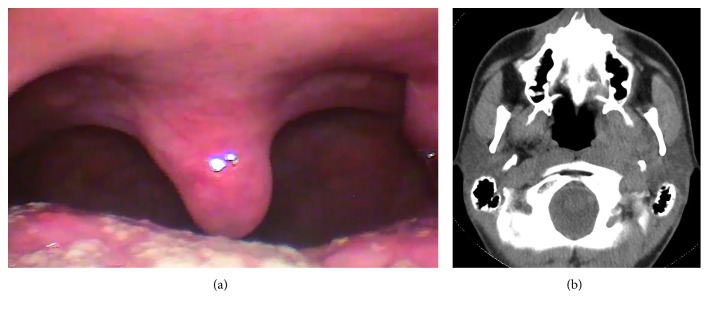
(a) Normal contour of the oropharynx six months postoperatively. (b) Computed tomography axial image: no tumor relapse is seen.

Six months postoperatively, no tumor relapse was observed during either physical examination or the repeated CT study (Figure 6).

Currently, the patient has been observed for a period of two years. There are no signs of schwannoma relapse.

## 3. Discussion

According to the literature, tumors of the parapharyngeal space are rare. It was reported previously that only 0.5 percent of all head and neck tumors are located in the parapharyngeal space [2]. Neurogenic tumors more often occur in the poststyloid compartment of the parapharyngeal space than in the prestyloid compartment [4]. In the majority of cases, cervical schwannomas are found in young and middle-aged people [2], while very rare in children [5].

Usually, schwannomas grow slowly and for a long time without causing any symptoms. However, a fully formed tumor causes pressure on the surrounding tissues. Clinical symptoms depend on the anatomy of the area of a growing tumor. Moreover, during visual inspection, a volumetric formation without mucositis in the lateral pharyngeal wall is observed. In the presented case, the redness of the pharyngeal mucosa was likely related to the previous multiple punctures. The diagnosis is confirmed by biopsy and histologic tumor examination. Histologically, schwannoma is a limited and well-encapsulated tumor occasionally presenting with a cystic degeneration component [6]. Such a cystic component was clearly delineated on the magnetic resonance image (Figure 3). Histologically, the tumor is differentiated from possible malignant tumors—fibrosarcomas, leiomyosarcomas, and fibrotic histiocytomas.

Radiological studies are very important for the diagnosis of the disease and surgical planning. Different authors [2, 7] recommend an initial evaluation of the tumor with contrast-enhanced CT examination, which was first performed in our case (Figure 2). MRI remains the “gold standard” for the diagnosis of these tumors. Radiological diagnostic methods can be used to identify the source-nerve of a growing tumor and to reduce the risk of postoperative nerve damage. Typical radiological diagnostic characteristics of schwannomas are the following: well-limited formation that deploys the surrounding structures without invading the surrounding tissue. Cystic and fatty degeneration, as well as signs of hemorrhage and calcification, can also be observed.

The preferred treatment of schwannomas is surgical in nature. Schwannomas are largely resistant to radiotherapy. Therefore, this method of treatment is of limited applicability. There are different removal techniques of the parapharyngeal space lesions: transoral, transcervical, transparotid, transcervical-transmandibular, and lateral skull base approaches. The transoral approach can only be used for benign prestyloid space tumors. However, this approach is considered to be unsafe since it is related with many postoperative complications such as hemorrhage, fistulas, and nerve damage [8]. A transcervical-transparotid approach identifies and preserves not only the facial nerve but also the external and internal carotid arteries, the internal jugular vein, the cranial nerves IX, X, XI, and XII, and the sympathetic nervous system chain. Most deep-lobe parotid gland tumors can be removed with this approach, and it is particularly effective for small tumors. The transcervical-transmandibular approach provides good control of tumor extension towards the skull base, the pterygomaxillary fossa, and large neck vessels [9]. We chose the transoral approach, since the larger part of the tumor was located in the prestyloid compartment of the parapharyngeal space. We also preferred this method since the patient has anatomically large oral cavity. Preoperatively, we discussed with the patient the possibility of the intraoperative switch to a transmandibular approach in case of insufficient space to remove the tumor totally. However, according to the literature data, transoral removal of the tumor might increase the risk of complications (nonradical tumor removal, massive bleeding, infection, and IX–XII cranial nerve damage), although no neurological complications or complications of other types were observed after the operation in the discussed case. In all cases, a radical removal of the tumor is recommended. After the removal of the tumor capsule, the risk of relapse is significantly reduced. When an external tumor removal technique is used, the tumor is accessed through the neck, parotid gland, cheekbones, lower jaw, the mastoid part of the temporal bone, and the infratemporal space, or a combination of these access methods is used [7]. After the removal of the tumor, the most common postoperative complication is vocal cord paralysis, which occurs in up to 85 percent of all the cases and results in hoarseness [7].

The literature data indicate that the prognosis of encapsulated schwannomas is good because radical removal of the tumor results in the full recovery of a patient. Relapses and malignant transformation of the tumor are very rare. If the tumor is removed nonradically, a re-excision is appropriate.

## 4. Conclusions

Schwannomas of the parapharyngeal space are very rare. The majority of patients experience painless pressure phenomena without neurological deficit. Computed tomography and magnetic resonance imaging studies are the main diagnostic methods to determine the exact diagnosis, and thus should be performed before planned management treatment of the tumor, which is usually radical removal of the tumor with its capsule.
